# Real-time, multi-spectral motion artefact correction and compensation for laser speckle contrast imaging

**DOI:** 10.1038/s41598-022-26154-6

**Published:** 2022-12-15

**Authors:** Wido Heeman, Hanno Maassen, Klaas Dijkstra, Joost Calon, Harry van Goor, Henri Leuvenink, Gooitzen. M. van Dam, E. Christiaan Boerma

**Affiliations:** 1 Faculty Campus Fryslân, University of Groningen, Wirdumerdijk 34, Leeuwarden, 8911 CE The Netherlands; 2 Department of Surgery, University Medical Centre Groningen, Hanzeplein 1, Groningen, 9713 GZ The Netherlands; 3 LIMIS Development BV, Henri Dunantweg 2, Leeuwarden, 8934 AD The Netherlands; 4 Department of Pathology and Medical Biology, University Medical Centre Groningen, Hanzeplein 1, Groningen, 9713 GZ The Netherlands; 5 Centre of Expertise in Computer Vision and Data Science, NHL Stenden University of Applied Sciences, Rengerslaan 8-10, Leeuwarden, 8917 DD The Netherlands; 6 ZiuZ Visual Intelligence, Stationsweg 3, Gorredijk, 8401 DK The Netherlands; 7 Department of Intensive Care, Medical Centre Leeuwarden, Henri Dunantweg 2, Leeuwarden, 8934 AD The Netherlands

**Keywords:** Biomedical engineering, Translational research

## Abstract

Laser speckle contrast imaging (LSCI) is so sensitive to motion that it can measure the movement of red blood cells. However, this extreme sensitivity to motion is also its pitfall as the clinical translation of LSCI is slowed down due to the inability to deal with motion artefacts. In this paper we study the effectiveness of a real-time, multi-spectral motion artefact correction and compensation by subduing an in vitro flow phantom and ex vivo porcine kidney to computer-controlled motion artefacts. On the in vitro flow phantom, the optical flow showed a good correlation with the total movement. This model results in a better signal-to-noise ratios for multiple imaging distances and the overestimation of perfusion was reduced. In the ex vivo kidney model, the perfusion overestimation was also reduced and we were still able to distinguish between ischemia and non-ischemia in the stabilized data whereas this was not possible in the non-stabilized data. This leads to a notably better perfusion estimation that could open the door to a multitude of new clinical applications for LSCI.

## Introduction

Laser speckle contrast imaging (LSCI) is based on the principle that backscattered light from a biological tissue, illuminated with coherent light, forms a random speckle pattern at the detector. It is a dye-free and full-field imaging technique that can measure subsurface microperfusion in tissue in real-time. LSCI was introduced in 1981 by Fercher and Briers to monitor blood flow^1^. Yet, LSCI has not become standard of care, in part due to movement artefacts^2^. Use of LSCI is based on the assumption that the dynamic change in the so-called speckle pattern contains information about blood flow. The random speckle pattern is the result of different optical path lengths and it is so sensitive to motion that the speckles are influenced by red blood cells (RBCs). However, this extreme sensitivity to motion is also its pitfall. Unwanted movement of the tissue of interest decreases the measured contrast, resulting in incorrect flow estimates (i.e., an overestimation of perfusion). This limits the use of LSCI in several clinical settings where motion cannot be eliminated, such as respiratory motion, pulsatile- and peristaltic- movement, uncontrolled movement in infants and elderly, and as a result of pain, and shivering.

Until now, efforts to reduce the effect of motion artefacts have mainly focused on the application of external information on movement, derived from an opaque plaque or the ECG signal. In these cases additional information has been used to mathematically correct for the influence of movement on LSCI^3–9^. However, these methods are not real-time and require the use of some sort of marker within the field-of-view. The latter of which is not always possible in some situations such as in a laparoscopic setting^10^.

In this study, we present a real-time and robust multi-spectral LSCI motion correction and compensation model, based on optical flow that could enable clinical translation of LSCI for the evaluation of microperfusion in various clinical working fields. We present our correction model for motion artefacts in two different experimental settings to demonstrate the basic concept up towards the pre-clinical application.

## Materials and methods

### Experimental setup

**Figure 1:**
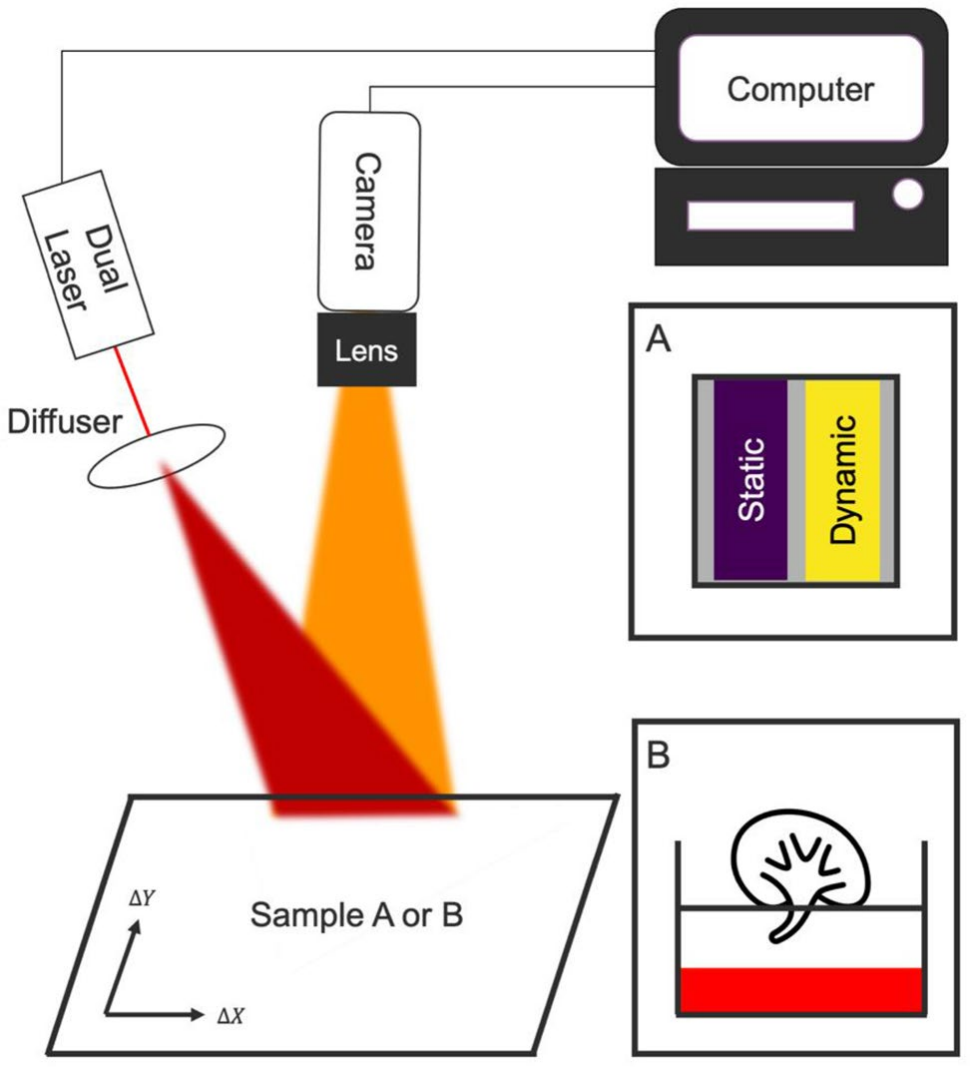
The dual-laser laser speckle contrast imaging (LSCI) setup with programmable 2D-movement artefact generator. The LSCI setup consisted of a color sensor, a computer and a dual-laser setup with a red 680 nm and green 532 nm 200mW laser. The samples a and b are placed on the 2D programmable motion platform. (**A**) The in vitro flow phantom presents one static- and one dynamic speckle region. (**B**) An ex vivo perfused porcine kidney.

We investigate the influence of controlled motion artefacts on the measured perfusion in two settings; an in vitro flow phantom and on an ex vivo perfused porcine kidney (Fig. 1). The perfusion measured by LSCI is expressed in laser speckle perfusion units (LSPU). The LSPU are calculated using Eq. (1) where $\sigma$ is the standard deviation of the intensity $I$ over the mean intensity $<I>$ using a spatial LSCI algorithm^11^. The spatial window was set to 5 × 5^12^ and the temporal window was set to 8 frames. Both the flow phantom and ex vivo perfused porcine kidney were subject to the same simulated motions. A programmable 2D movement platform with 4 different pathlengths at 5 different speeds ranging from 4—12 mm/s in increments of 2 mm/s was used. The architecture is a 2-axis movement system commonly found in 3D-printers with two servomotors that are controlled by a microcontroller (Mega 2560 REV3, Arduino, Ivrea, Italy). Compared to Ambrus *et al.*^13^ this setup has the advantage that speed, distance and direction can be programmed and controlled by the user and thus imitate biologically relevant motion artefacts.

1 $$LSPU = \frac{1}{K}=\frac{<I>}{\sigma }$$

The first setting investigated the influence of motion artefacts in vitro on a flow phantom with one stable dynamic- and one stable static- speckle region (Fig. 1a) (FLPI Cal 2FPS, Moor Instruments Ltd, Millwey, United Kingdom). This flow phantom provides an optimal setting for experimenting with the motion correction and compensation, due to extremely clear and distinct optical features such as the written text. It has two 20- by 10-mm tubes that present itself as dynamic and static speckle areas. This allows for isolation of the effect of motion artefacts using the stable speckle contrast $K$ (Eq. 1). We have calculated the signal-to-noise ratio (SNR) between the region-of-interest (ROI) covering the static speckle region (LSPU^static^) and the ROI covering the whole dynamic speckle region (LSPU^Dynamic^) using Eq. (2). SD^Static^ and SD^Dynamic^ are the standard deviation of the LSPU^static^ and LSPU^Dynamic^ respectively. The LSPU and SD values are calculated for each frame where the ROI automatically tracks the static and dynamic regions on the flow phantom using the stabilization algorithms. The SNR is indicative of the ability to distinguish between well- and poorly- perfused tissue. The distance between the camera and flow phantom was 90 or 200 mm to examine the effect of the motion-to-pixel ratio.

2 $$SNR=\frac{{LSPU}^{Dynamic}-{LSPU}^{Static}}{{SD}^{Dynamic}+{SD}^{Static}}$$

The perfusion overestimation is calculated by comparing the LSPU during movement compared to the LSPU when the flow phantom is stationary. These values will be compared between the stabilized and non-stabilized LSCI algorithm. Also using this model, we will investigate if optical flow is indicative of motion artefacts. This will be studied by looking at the correlation of the total optical flow and the movement time and the correlation between optical flow and LSPU. In order to investigate the use of optical flow as an indication of motion artefacts we study the relationship between LSPU values of frames that have an optical flow equal to zero compared to an optical flow of larger than zero for both the static and the dynamic region of the flow phantom. Optical flow is a displacement vector that can be used without the need for a fiducial marker in the field-of-view (FOV). The accuracy of the optical flow detection is highly dependent on the quality of the distinct optical features. We calculate the optical flow between every subsequent frame. The accuracy of optical flow for geometrical realignment has been proven before^14^. As mentioned by others^7^, any motion will falsely increase the LSPU value from the true perfusion value. Since the flow phantom is not subject to uncontrollable hemodynamic changes, all deviations in LSPU must be the result of motion. Hence, there should be a correlation between optical flow increased LSPU values.

The second experiment investigated the influence of motion artefacts using the programmable platform on an ex vivo machine perfused porcine kidney (Fig. 1b). This adds complications such as the monitoring of a hemodynamic event, small tissue vibrations and specular reflections. The slaughterhouse retrieved porcine kidneys were obtained from a local abattoir. Pigs (female Dutch landrace pigs, approximately five months old with an average weight of 130 kg) were slaughtered for consumption purposes and were handled according to standardized legal procedures. The ex vivo machine perfused porcine kidneys setup was described in detail previously^15^. Two different experiments were repeated three times on one kidney during normothermic machine perfusion. First, controlled movement artefacts were generated during stable pressure and stable hemodynamic state. Second, we induced a local perfusion deficit by inflating a balloon catheter in the renal artery. LSCI measurements with and without movements were made in the presence of ischemia in the inferior lobe of the kidney. Similar to the flow phantom, the perfusion overestimation is calculated by comparing the LSPU during movement compared to the LSPU when the kidney is stationary.

### Laser speckle contrast imaging setup

The LSCI setup consisted of a color camera, a fiber coupled dual-laser module with optics and a laptop computer (Fig. 1). The multi-spectral setup was chosen to create trackable distinct optical features for the optical flow calculation. The hypothesis is that the green laser, on most tissues, gives rise to these visually distinct features around blood vessels since these appear as dark veins. The red laser is used for perfusion measurements. The color camera was a 12-bit, 4.19-megapixel (5.5-$\upmu$m $\times$ 5.5-$\upmu$m pixel size) CMOS camera (UI-3370CP-C-HQ, IDS Imaging Development Systems GmbH, Obersulm, Germany), equipped with a 12.5 mm, F1.5–16 lens (LM12HC, Kowa, Dusseldorf, Germany). The images were acquired at 40 ms exposure time, at a rate of 25 Hz with the f-stop set to 8. The relatively long exposure time is required in order to get adequate pixel intensities as a result of the combination of a low powered laser and large FOV. The set imaging parameters resulted in a minimum of ~3 pixels per speckle, thus satisfying the Nyquist criterion^16^. The multi-spectral stabilization requires two laser diodes, we chose to use a red ($\uplambda$ = 680 nm, 200 mW, Lionix International, Enschede, The Netherlands) and green ($\uplambda$ = 532 nm, 200 mW, Lionix International, Enschede, The Netherlands) fiber coupled laser diode that were coupled into a fiber port (Thorlabs, Newton, United States), with a collimating lens (12 mm $\varnothing$, -12 mm FL uncoated double-concave lens (Edmund Optics, New Jersey, United States) placed at a distance of 40 mm to fully illuminate the FOV. The crosstalk was 5% of the 532 nm in the red channel and 4% of the 632 nm into the green channel respectively. The camera and laser were mounted 9 cm and 20 cm above and perpendicular to the in vitro flow phantom resulting in an imaging FOV of 80 × 80 mm and 200 × 200 mm respectively. The imaging distance was 200 mm for the ex vivo measurements resulting in a 200 × 200 mm FOV. The camera is connected to a laptop computer (Dell XPS 15, Intel core i7-8750H CPU, 16 GB Ram, GeForce GTX1050 Ti GPU) that is loaded with Lapvas-Imaging software (LIMIS Development BV, Leeuwarden, The Netherlands).

### Movement artefact correction and compensation model

The technology for the detection of perfusion (i.e. LSCI) is based on the scattering of photons on red blood cells (RBCs)^17^. When perfusion is measured in tissue that is subject to even the slightest of motion, the measured perfusion values consist of the movement of RBCs (wanted) and the motion artefact due to the movement of the tissue (unwanted). We hypothesized that the traditional LSCI value $K$ (Eq. 1) is comprised of the movement of the RBCs ($fRBC$) and the unwanted movement of the tissue ($fTIS$) as in Eq. (3). The proposed solution is to minimize the influence of $fTIS$ in $K$ to more accurately estimate $fRBC$. This is split up into two parts; a motion artefact compensation based on sudden changes in LSCI values and the geometrical alignment based on the use of optical flow-derived displacement vectors. The stabilized images were displayed to the researchers at 14 frames per second with a processing speed of 70 ms per image on the laptop computer.

3 $$K=fRBC+fTIS$$

#### Motion artefact compensation

Images affected by motion artefacts have a decreased SNR and to a certain degree a decrease in image quality. It is not possible to separate $fRBC$ and $fTIS$ from $K$. However, it is possible to estimate the relative effect that $fTIS$ has on $K$. A sudden change in $K$ is likely the effect of $fTIS$, since most perfusion measurements are performed in a stable hemodynamic state. Weights based on sudden differences or changes in speckle contrast, may be indicative for unwanted motion artefacts. Hence, applying these weights inverse to the average LSPU might result in a higher image quality. The use of weight factors inversely correlated to the relative change in $K$ compared to the average $K$ of the previous images can in this way compensate the effect of the motion artefact $fTIS$ on $K$. The temporal weight factor ${w}_{t}$ of pixel $(i,j)$ is calculated using Eq. (4). The weight factors are divided by the sum of all weight factors in the temporal window (i.e., linear normalization) resulting in ${w}_{t\left(normalized\right)}$. Subsequently, the normalized weight factor is used for the multiplication of the temporal LSPU for each pixel.

4 $${w}_{t}\left(i, j\right)=\frac{1}{{LSPU}_{t}\left(i,j\right)}$$

Lastly, we hypothesized that most disturbances of $fTIS$ are present in a recursive pattern such as respiratory motion and heartbeat. To minimize the influence of these unwanted motion artefacts the temporal window should be the size of at least twice the slowest recursive motions. By failing to adhere to the Nyquist criterion, the average noise will not be the same if the temporal window is smaller than the slowest recursive. When the temporal window used in the spatial LSCI is large enough the window will always encompass the same amount of noise.

#### Geometrical alignment based on optical flow

Due to motion of the tissue and camera, images become misaligned, therefore, geometrical alignment is required. The proposed geometrical alignment is based on the use of optical flow. The sparse pyramidal Lucas-Kanade method is used for the displacement vector calculations^18^. Optical flow is calculated using the positions of automatically determined corresponding features in two subsequent images on which the displacement vectors identifying the motion of the target area are determined. The features are detected by the sparse pyramidal Lucas-Kanade algorithm. The optical flow values are equal to the number of pixels that a detected feature moves between two subsequent images. The transformations based on the displacement vectors are then used to register or geometrically align the speckle contrast images. The geometrical alignment consists of an affine transformation determined by the optical flow vectors.

We have designed a multi-spectral two laser setup in order to generate visually distinct object features in perfused tissue. Superficial blood vessels absorb green light and appear darker. This results in an increased contrast that in turn increases the salient features in the image that can be tracked. The optical flow is calculated based on solely the green pixels.

### Data analysis

Graphpad Prism (Prism 7, La Jolla, United States of America) was used for statistical analysis. All data are presented as mean ± standard deviation. A linear regression test and Welch’s T-tests were used. A two-sided p-value < 0.05 was considered statistically significant.

## Results

### Optical flow is indicative of motion artefact

**Figure 2:**
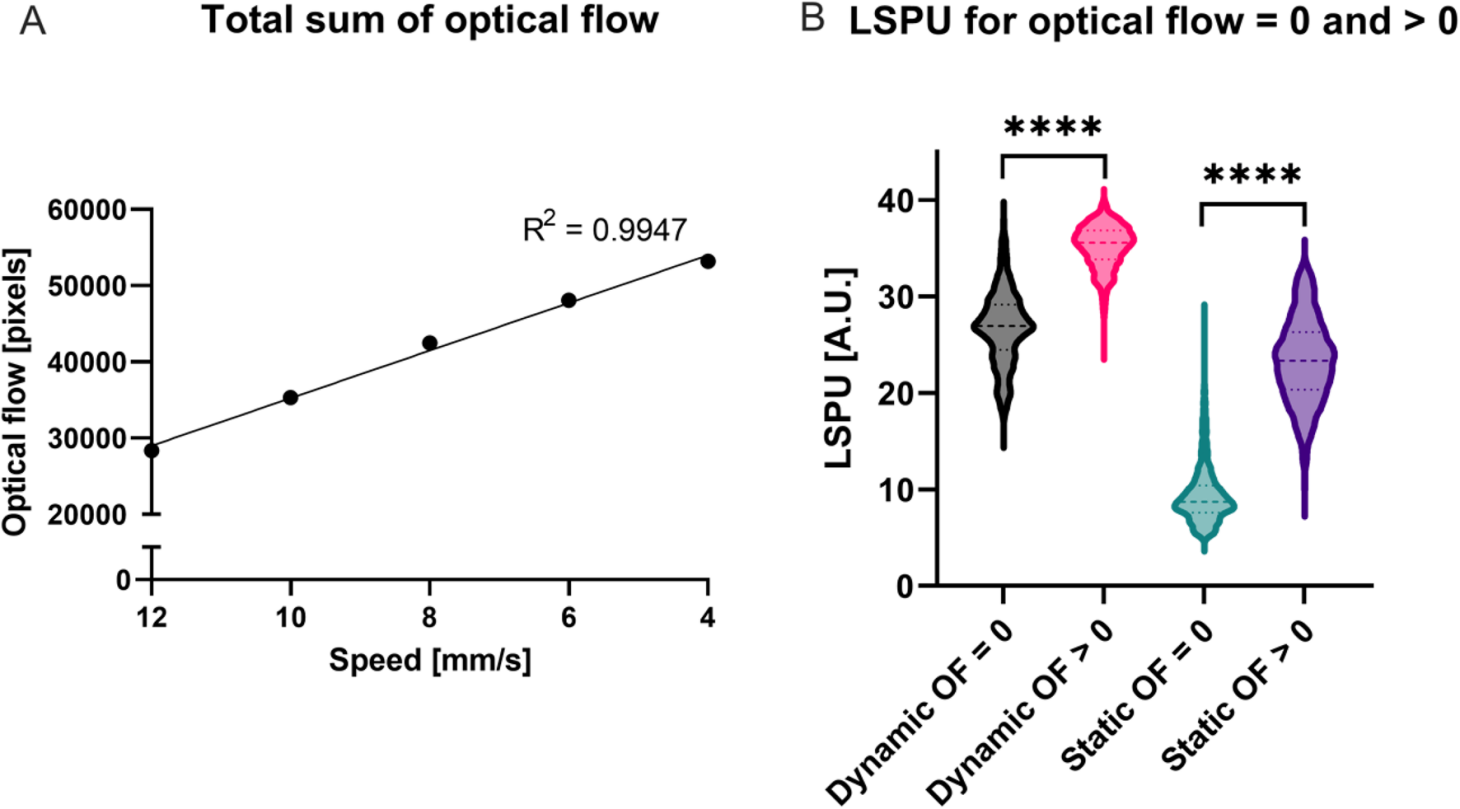
(**A**) Sum of optical flow measured per movement speed in mm/s with a linear fit. The R-squared for a linear fit is 0.9947. (**B**) A box-and-violin plot of the laser speckle perfusion units (LSPU) sorted by the measured optical flow (OF). To investigate the use of optical flow as an indication for motion artefacts, we studied the relation between OF and non-stabilized LSPU. LSPU sorted by OF values of = 0 and > 0. **** are significantly different (p < 0.0001).

To investigate the use of optical flow as an indication for motion artefacts, we studied the relation between optical flow and non-stabilized LSPU. As can be seen from Fig. 2a, the optical flow increases linearly with the increase in total movement time for the phantom (R^2^ = 0.9947). This was in line with the fact that total movement time also increases linearly. We have studied the effect of motion on the static and dynamic regions of the phantom. When looking at the LSPU average values of the static region that are associated with an optical flow of equal to zero, we see that there was a significant difference in LSPU compared to an optical flow of > 0 (p < 0.0001) (Fig. 2b). This is the same for the LSPU of the dynamic region (p < 0.0001) (Fig. 2b).

### In vitro flow phantom motion artefact correction and compensation

**Figure 3:**
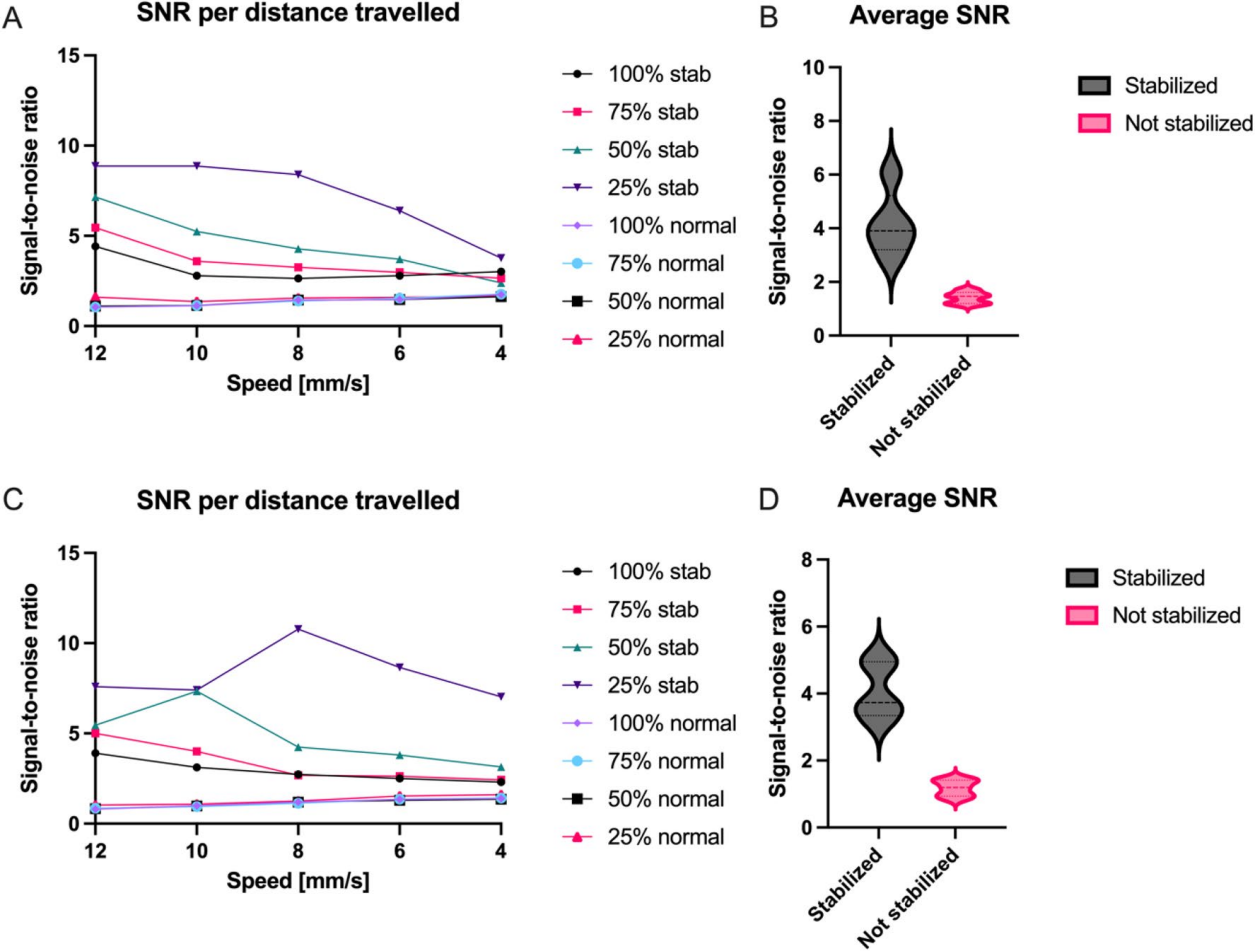
(**A**) Signal-to-noise ratio for each distance per movement speed in mm/s for the 20-cm camera distance. (**B**) Box-and-violin plots of average signal-to-noise ratios for the 20-cm camera distance. (**C**) Signal-to-noise ratio for each distance per movement speed in mm/s for the 9-cm camera distance. (**D**) Box-and-violin plots of average signal-to-noise ratios for the 9-cm camera distance.

**Figure 4:**
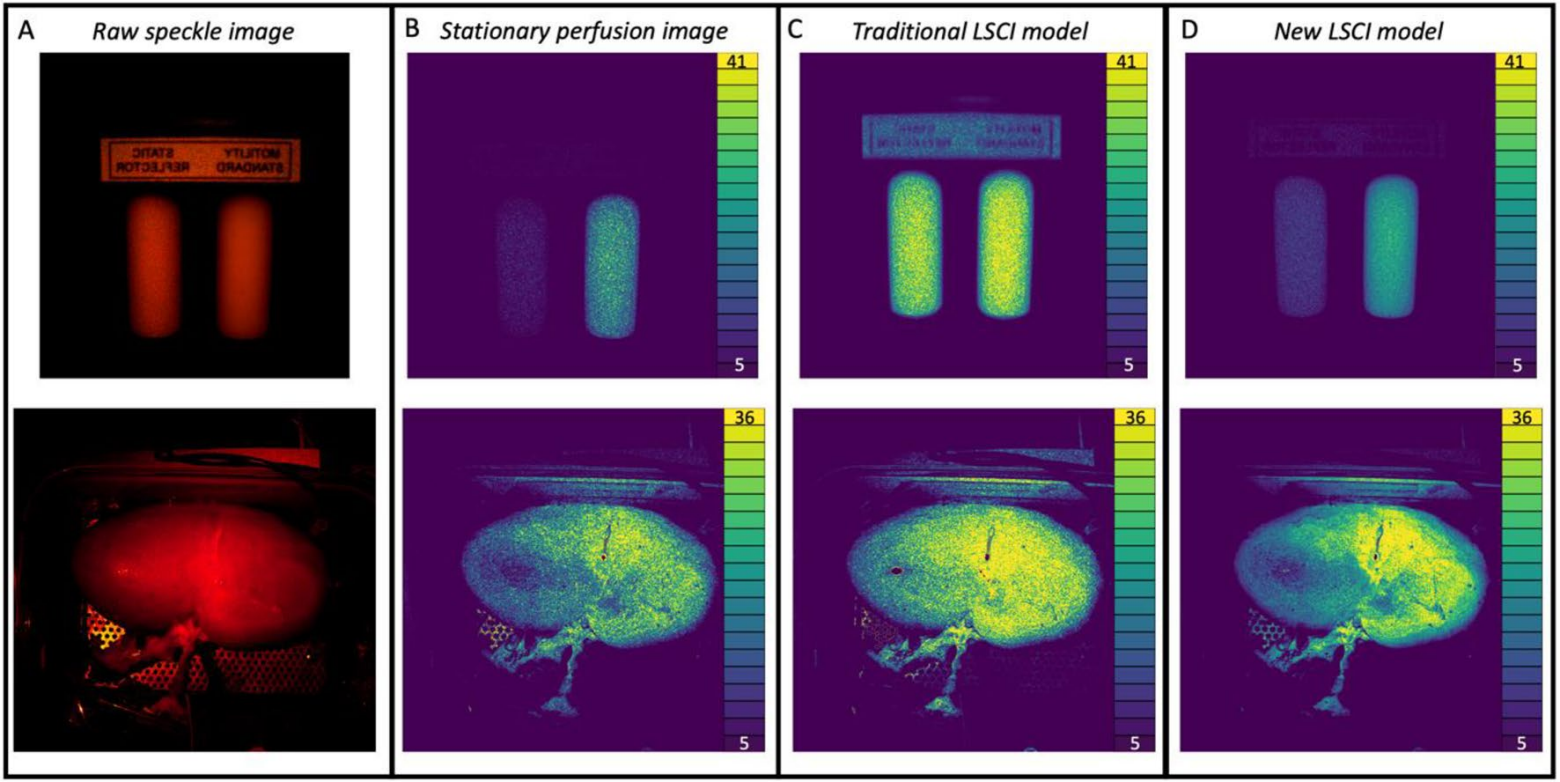
(**A**) The unprocessed image of the flow phantom (top) and ex vivo kidney (bottom). (**B**) Perfusion images of the stationary flow fantom (top) and ex vivo kidney (bottom) displayed in laser speckle perfusion units (LSPU) [A.U.]. (**C**) Perfusion image (the same as **D**) of non-stationary flow phantom (top) and ex vivo kidney (bottom) using the traditional laser speckle contrast imaging (LSCI) mode in LSPU [A.U.]. D: Perfusion image (the same as **C**) of non-stationary flow phantom (top) and ex vivo kidney (bottom) using the multi-spectral motion correction and compensation LSCI model in LSPU[A.U.].

A higher SNR indicates a high discriminative power to distinguish between well- and poor perfused tissue. The SNRs for the 20-cm camera distance are found in Fig. 3a. The average SNR (and standard deviation) was 4.15 ± 1.21 for the stabilized versus 1.41 ± 0.22 non-stabilized, meaning an increase of the SNR by a factor of 2.94. (Fig. 3b). The smaller 9-cm camera distance performed slightly better as the SNR was 3.44 times higher. The SNRs are found in Fig. 3c and the average SNR are with a stabilized SNR of 4.06 ± 0.84 and a non-stabilized SNR of 1.18 ± 0.24 (Fig. 3d). Figure 4 shows an example of the stationary (Fig. 4a,b) and non-stationary flow phantom perfusion images using the traditional—(Fig. 4c) and stabilized (Fig. 4d) LSCI algorithms.

We also see a large decrease in the standard deviation of stabilized recordings, especially in the static speckle tube. By nature, the standard deviation is higher for static speckles. We see an increase of the SNR by a factor of 3.05 (SD = 2.46 vs SD = 7.52) for the static speckles and a 2.33 (SD = 1.75 vs SD = 4.09) for the dynamic speckles. This is similar for the smaller camera distance with 3.43 (SD = 2.23 vs SD = 7.64) for the static and 2.43 (SD = 1.95 vs SD = 4.75) for the dynamic speckles.

For the larger camera distance, these numbers translate to 7.31 times reduction in perfusion overestimation (50.86% vs 6.96%) and 5.65 times reduction in overestimation for static speckles (90.93% vs 16.09%). This effect is less prominent for the smaller camera distance with 2.79 times less reduction in perfusion overestimation (44.84% vs 16.09%) and 4.81 for the static speckles (124.86% vs 25.98%).

### Ex vivo perfused porcine kidney model motion artefact correction and compensation

In concordance with the linear increase in total movement time we observed a linear increase in optical flow (R^2^ = 0.8375 ± 0.0957) in the ex vivo perfused porcine kidney setup. The static hemodynamic experiment showed a noise reduction of 1.62 times in the standard deviation of stabilized recordings (SD = 1.64 ± 0.32) compared to the non-stabilized data (SD = 2.67 ± 0.23). We also observed that the average overestimation of perfusion was 11.21 ± 3.03% compared to 26.96 ± 2.89%, i.e., a 2.40 times improvement. Figure 4 shows an example of the stationary (Fig. 4a,b) and non-stationary kidney perfusion images using the traditional—(Fig. 4c) and stabilized (Fig. 4d) LSCI algorithms.

**Figure 5:**
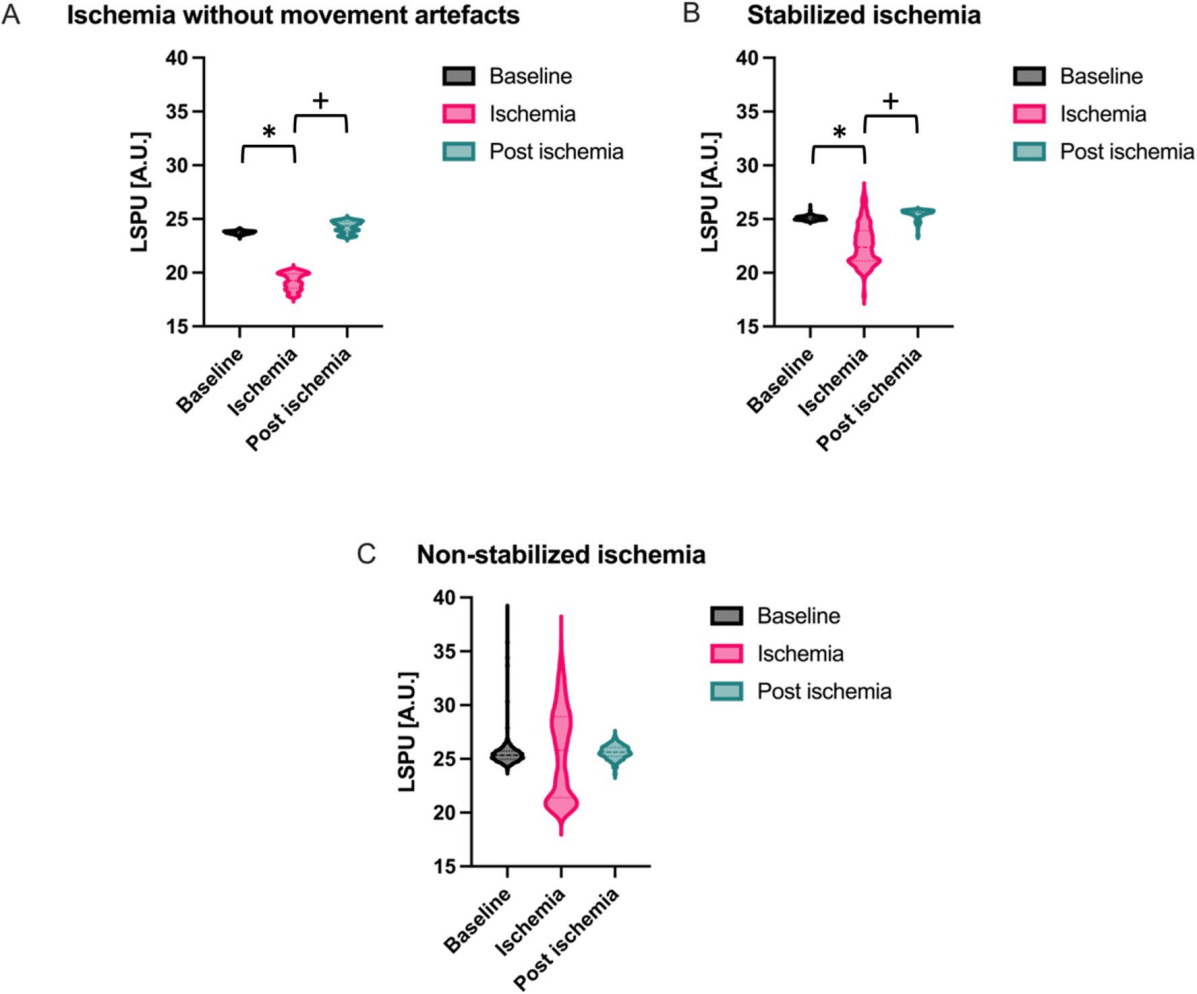
(**A**) Box-and-violin plot of baseline, ischemia and post-ischemia LSPU of local ischemia experiment without movement artefacts. (**B**) Box-and-violin plot of baseline, ischemia and post-ischemia stabilized LSPU of local ischemia experiment with movement artefacts. (**C**) Box-and-violin plot of baseline, ischemia and post-ischemia non stabilized LSPU of local ischemia experiment with movement artefacts. * = ischemia is significantly different from baseline. +  = Post occlusion is significantly different from ischemia.

By inflating a balloon catheter, we induced local ischemia in one of the bifurcations of the renal artery, creating a high flow and low flow area, comparable to the flow phantom as described above. The results are depicted in Table 1. The ischemia was induced once without- and twice with- the addition of movement artefacts. We observed that, although there is more noise compared to the non-moving local ischemia (Fig. 5a), we could still distinguish between ischemia and non-ischemia in the stabilized data (p < 0.0001) (Fig. 5b) whereas this was not possible in the non-stabilized data (p > 0.05) (Fig. 5c).

**Table 1:** The average measured laser speckle perfusion units (LSPU) compared to baseline calculated over the ischemic and post-ischemic periods for the ischemia without movement artefacts, for the stabilized ischemia and the non-stabilized ischemia.

	Ischemia without movement artefacts	Stabilized ischemia	Non-stabilized ischemia
Baseline	100.00%	100.00% ± 0.00%	100.00% ± 0.00%
Ischemia	81.33%	82.70% ± 0.96%	86.40% ± 1.24%
Post ischemia	99.74%	100.88% ± 0.91%	99.75% ± 1.99%

## Discussion

In this paper we report on a real-time motion artefact correction as a compensation model for the clinical evaluation of subsurface microperfusion using LSCI. Our data show that it is possible to significantly reduce the effect of motion artefacts in real-time. We validated the model in vitro using a stable flow phantom as a most ideal model. We concluded from this experiment that optical flow is indicative for motion artefacts and that it can be used for image alignment. We have seen a significant improvement in SNRs and a significant decrease in standard deviation. This leads to a notably better perfusion estimation in comparison to the non-stabilized model. The ex vivo perfused porcine kidney added substantial challenges, such as tissue vibrations and specular reflections. The most important finding is that by using this stabilization model we still had the ability to distinguish between well- and non-perfused tissue. We believe that qualitative perfusion assessment could be helpful to the acceptance of LSCI in the clinical setting. Our results suggest that the motion artefact correction technology can be applied in a multitude of new clinical applications.

The movement artefacts induced in this study are larger than artefacts found in clinical practice. Yet, the model was still able to significantly improve the LSCI data. The decrease in SNR that can be found with a decrease in movement speed and increasing distance substantiate that low frequency, longer lasting continuous artefacts lessen the models effect compared to high frequency and shorter artefacts. This is caused by the algorithms inability to know the “true” flow estimate when a movement is takes longer than the length of the temporal window. The model should thus be finetuned for typical movements present in the clinical setting by changing the temporal window. On the other hand, the algorithm cannot detect the motion when the movements are faster than the framerate. This should also be explored for each clinical application separately.

We have chosen not to rely on one source of information by using the sudden changes in LSPU for the compensation and the optical flow for the correction. Since we have shown that optical flow is indicative of motion artefacts and that there is a significant difference in LSPU for optical flow values of zero and lager than zero, other combinations might be feasible. For example, by using a weighted combination of optical flow and sudden change in LSPU for the motion compensation.

The advantages of the method presented in this manuscript compared to previous works is that this method is real-time and without the need of any form of fiducial marker or opaque adhesive plaque. We foresee that this model can help with the clinical acceptation of LSCI as a qualitative and semi-quantitative perfusion imager. This study specifically could serve as a pre-clinical study for intraoperative perfusion measurements during kidney transplantation. The real-time 2D-perfusion maps could help with the early detection of local perfusion deficits after reperfusion of the kidney, that could ultimately lead to better post-operative outcomes. The ability to correct for motion might improve burn wound imaging, where patients often shiver in pain. Moreover, our group has embedded this model in a laparoscopic formfactor with the intention of monitoring colonic perfusion. The laparoscopic formfactor might have more applications in fields such as upper gastro-intestinal surgery and other laparoscopic interventions.

Our study limitation is that we only validated this model in the most ideal setting and ex vivo on a kidney. However, the most optimal multi-spectral setup should be explored for each clinical indication as different tissue optical properties and biological properties might affect the distinct optical features. The 14 frames per second framerate of the stabilized images can probably also be improved by image processing optimization and upgraded hardware with more processing power. Future efforts should be made regarding pre-clinical research for other tissue types. Also, the multi-spectral correction could be improved by replacing the green coherent light source with a narrow spectral bandwidth LED as this minimizes unwanted speckle noise without introducing unwanted crosstalk.

## Conclusion

In summary, our in vitro and in vivo data suggests that this LSCI stabilization model is effective in the reduction of the effect of motion artefacts, enabling the distinguish between well- and poor perfused tissue during substantial movement of the region of interest. This method might increase the clinical applicability of LSCI, since it is suitable for most clinical indications as there is no need for placement of markers in the field of view and the processing can be done in real time.

## Data Availability

The datasets used and/or analyzed during the current study available from the corresponding author on reasonable request.
